# Network Pharmacology-Based Study on the Mechanism of *Pinellia ternata* in Asthma Treatment

**DOI:** 10.1155/2020/9732626

**Published:** 2020-10-20

**Authors:** Yanmin Lyu, Xiangjing Chen, Qing Xia, Shanshan Zhang, Chengfang Yao

**Affiliations:** ^1^Institute of Basic Medicine, Shandong First Medical University (Shandong Academy of Medical Sciences), 18877 Jingshi Rd, Jinan 250062, Shandong, China; ^2^The First Clinical Medical College, Shandong University of Traditional Chinese Medicine, Jinan 250000, Shandong, China; ^3^Biology Institute, Qilu University of Technology & Shandong Academy of Sciences, Jinan 250014, Shandong, China

## Abstract

**Background:**

*Pinellia ternata* (PT), a medicinal plant, has had an extensive application in the treatment of asthma in China, whereas its underlying pharmacological mechanisms remain unclear.

**Methods:**

Firstly, a network pharmacology method was adopted to collect activated components of PT from Traditional Chinese Medicine Systems Pharmacology Database and Analysis Platform (TCMSP). Targets of PT were assessed by exploiting the PharmMapper website; asthma-related targets were collected from the OMIM website, and target-target interaction networks were built. Secondly, critical nodes exhibiting high possibility were identified as the hub nodes in the network, which were employed to conduct Gene Ontology (GO) comment analysis and Kyoto Encyclopedia of Genes and Genomes (KEGG) signaling pathway enrichment analysis. Finally, the tissue expression profiles of key candidate genes were identified by the Gene Expression Omnibus (GEO) database, and the therapeutic effect of PT was verified by an animal experiment.

**Results:**

57 achievable targets of PT on asthma were confirmed as hub nodes through using the network pharmacology method. As revealed from the KEGG enrichment analysis, the signaling pathways were notably enriched in pathways of the T-cell receptor signaling pathway, JAK-STAT signaling pathway, and cytokine-cytokine receptor interaction. The expression profiles of candidate genes including *Mmp2, Nr3c1, il-10, il-4, il-13, il-17a, il-2, tlr4, tlr9, ccl2, csf2*, and *vef*g*α* were identified. Moreover, according to transcriptome RNA sequencing data from lung tissues of allergic mice compared to normal mice, the mRNA level of *Mmp2* and *il-4* was upregulated (*P* < 0.001). In animal experiments, PT could alleviate the allergic response of mice by inhibiting the activation of T-helper type 2 (TH2) cells and the expression of *Mmp*2 and *il-4*.

**Conclusions:**

Our study provides candidate genes that may be either used for future studies related to diagnosis/prognosis or as targets for asthma management. Besides, animal experiments showed that PT could treat asthma by regulating the expression of *Mmp2* and *il-4*.

## 1. Background

Asthma refers to an inflammatory disorder of the airways which is induced by common aeroallergens (e.g., house dust mites, fungi, and air pollutants) and characterized by airway hyperresponsiveness, variable airflow limitation, and mucous secretion, as well as chronic inflammation [1]. In asthma, there exists selective expansion of T lymphocytes (particularly of TH2 cells) that secrete a cluster of cytokines such as interleukins (IL)-2, IL-4, IL-5, IL-9, and IL-13 and granulocyte-macrophage colony-stimulating factor (GM-CSF), leading to mast cell differentiation and maturation, eosinophil maturation and survival, basophil recruitment, B cell differentiation, and production of IgE, which jointly orchestrate the allergic inflammatory cascade [2, 3]. On the whole, inhaled corticosteroid (ICS) or ICS combined with long-acting *β*2-adrenergic agonists has been the common clinical treatment to mitigate asthma. In some cases, however, corticosteroid overload raises a high risk of glucocorticoid-related adverse events and huge difficulty in controlling the onset of asthma [4]. For the asthma incidence, a single-ingredient medicine cannot satisfy the existing treatment of asthma, and there is an urgent need for practical, multitarget drugs to treat asthma that can remedy the deficiencies exhibited by those that are currently available.

**Figure 1:**
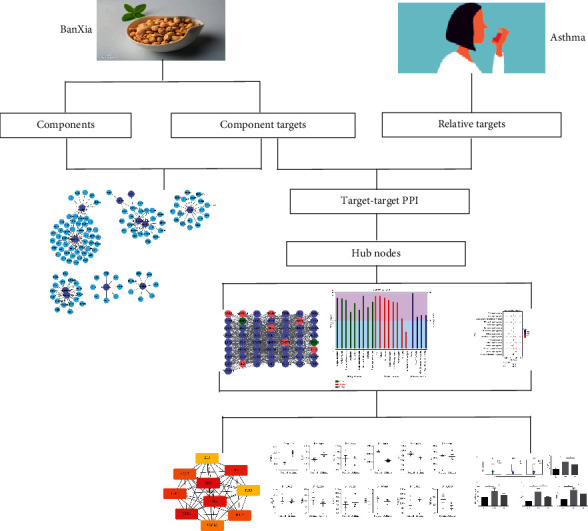
The schematic illustration of network pharmacology analysis and animal experimental verification for exhibiting pharmacological pathways of PT against asthma.

Traditional Chinese medicine (TCM) has been indispensable to the health of China. *Pinellia ternata*, also known as BanXia in Chinese, has been a common and potent medicinal herb in TCM practice. It has been clinically applied for suppression of the cough center to exert antitussive effects, facilitate cell division, decrease the viscosity of whole blood, and reduce inflammation, in conjunction with other herbs. However, the detailed mechanisms of their operation at molecular levels are still unknown, making it difficult in the design of more efficient and less toxic drugs for treatment. Thus, comprehensive and appropriate strategies are urgently required to gain deeper insights into how herbal formulae impact diverse biological processes in the treatment of diseases. Network pharmacology refers to an efficient and robust method having been employed extensively for several decades to treat a range of diseases, as coupled with high-throughput group analysis and virtual computing, as well as network database retrieval [5, 6]. Using network pharmacology in search of TCM is conducive to identifying the relationships between drug and disease, discovering active ingredients, elucidating the mechanism of action, and assessing drug safety [7]. In this study, a network pharmacological research was conducted to delve into practical components and action objectives of PT as an attempt to explore the underlying mechanisms of action of asthma treatment; furthermore, the therapeutic effect of PT was verified by OVA-induced allergic mice, and the regulation of key proteins was also examined (Figure 1).

## 2. Materials and Methods

### 2.1. Animal Experiments

#### 2.1.1. Mice Model

All animal experiments were approved by the Institutional Animal Care and Use Committee of the Shandong Academy of Medical Sciences. Female C57BL/6 mice, aged 7-8 weeks, were provided by Shandong Laboratory Animal Facility (Shandong Academy of Medical Sciences, Jinan, China). All animals were housed and bred in a temperature- and light-controlled environment under a 12 h light-dark cycle, and they were maintained under specific pathogen-free (SPF) conditions. Subsequently, mice were randomly split into three groups (the control group, the model group, and the herb group) covering four mice, respectively. The model group and herb group were injected intraperitoneally (i.p.) with 50 *μ*g OVA (Sigma-Aldrich, MO, USA) and 2.25 mg adjuvant aluminum hydroxide (Thermo Scientific, Pittsburgh, PA, USA) in a total volume of 100 *μ*L on day 0, 7, and 14. On day 21, the mice were intranasally (i.n.) administrated with 100 *μ*g OVA in 50 *μ*L [8, 9]. The herb group was administrated with PT (1.95 g/kg/d) (provided by Affiliated Hospital of Shandong University of Traditional Chinese Medicine, China) water decoction for 21 days, whereas the control mice were given the identical volume of phosphate-buffered saline (PBS). All mice were prepared for tissue harvesting.

#### 2.1.2. Flow Cytometric Staining

Lungs were cut into small pieces and then incubated with 1 mg/ml collagenase D (Sigma-Aldrich, MO, USA) for 30 min and were digested into single-cell suspension. Rat anti-mouse antibodies cover APC-CD45, PE-Cy7-CD3, PerCP-CD4, and PE-IL-4 (BD Biosciences, CA, USA). Intracellular cytokine staining was performed following the previous description [10]. Flow cytometric (BD FACSVerse™, BD Biosciences, USA) data were analyzed with FlowJo software.

#### 2.1.3. Measurement of IL-4 in Bronchoalveolar Lavage Fluid (BALF)

24 h after the final OVA challenge, all mice were sacrificed to prepare BALF. Lungs were lavaged in situ with 1 mL of ice-cold phosphate buffer solution (PBS) via the tracheal cannula. After three times lavage, approximately 2 mL of BALF was recovered and centrifuged at 4°C (1500 r/min) for 10 min [11]. The supernatants were rapidly collected and frozen for further cytokine measurement by ELISA. The concentration of IL-4 in BALF was measured using a specific mouse ELISA kit (R&D Systems, Minn., USA). ELISA experiments were performed according to the manufacturer's instructions.

#### 2.1.4. Reverse Transcription- (RT-) qPCR

Total RNA was extracted with TRIzol (CW.BIO, Beijing, China) from the lung tissue of mice. The concentrations of RNA were determined before reverse transcription. Total RNA (200 ng) was further reserve-transcribed to cDNA, and detail operation steps were done as previously described [9]. The primers used in the present study were as follows: for *Mmp2*, sense CAAGTTCCCCGGCGATGTC and antisense TTCTGGTCAAGGTCACCTGTC; for *il-4*, sense GGTCTCAACCCCCAGCTAGT and antisense GCCGATGATCTCTCTCAAGTGAT. Glyceraldehyde-3-phosphate dehydrogenase (GAPDH) was used as an endogenous control, and the comparative threshold cycle (2−ΔΔCT) equation was used to calculate the relative expression levels.

### 2.2. Screening Bioactive Components of PT

The components' data of PT originated from the TCMSP systems pharmacology database (http://lsp.nwu.edu.cn/tcmsp.php) [12]. To achieve the bioactive components' screening of PT in pharmacodynamics studies, two critical indicators have been usually taken as the screening criteria in ADME processes (e.g., absorption, distribution, metabolism, and excretion) and drug design, which include oral bioavailability (OB) and drug similarity (DL), and components were retained only if OB was over 30% and DL was more than 0.18 to satisfy criteria [13]. In this study, based on the relevant literature and the PubChem network database (https://pubchem.ncbi.nlm.nih.gov/), 11 bioactive components of PT were retained, and their corresponding information (PubChem ID, OB, and DL) was also acquired for subsequent analysis [14].

### 2.3. Component-Related Target Proteins' Prediction

To ascertain the relevant targets of the bioactive components in PT, the PharmMapper database (http://lilab.ecust.edu.cn/pharmmapper/get.php) was adopted [15]. In brief, for a given small molecule, potential candidate targets were identified using the reverse pharmacodynamic profiling method. Through the retrieving process, 115 assessed targets were screened out after duplicates were deleted, and they were made to conform to the correct UniProt ID. Component-target network maps were plotted by Cytoscape 3.7.2 for these data [16]. Subsequently, GO enrichment and KEGG network pathway analyses were conducted on the targets acquired by using the Database for Annotation, Visualization, and Integrated Discovery (DAVID, https://david.nicifcrf.gov/, updated in Mar. 2017) online [17]. Values of *P* < 0.05 were considered exhibiting statistical significance.

### 2.4. Asthma-Related Targets' Prediction

Data on the asthma-related targets were acquired from the Online Mendelian Inheritance in Man database (OMIM, updated December 21, 2019) [18, 19], a comprehensive, authoritative compendium of human genes and genetic phenotypes, which is freely available and daily updated. The keyword was “asthma,” and 259 asthma-related targets were harvested jointly.

### 2.5. Protein-Protein Interaction (PPI) Network Construction

To delve into the interaction between target proteins from a systematic and holistic perspective, PPI network mapping was conducted with STRING (http://string-db.org) [20]. Accordingly, the “component-target-disease” network was established to express the relationships between the targets corresponding to PT and interacting protein; furthermore, such network was visually analyzed with Cytoscape 3.7.2 software. As critical parameters of network analysis, the degree of centricity (DC), betweenness centrality (BC), close to the central (CC), the network centricity (NC), and local edge connectivity topology selection (LAC) were adopted to obtain the significance of the respective target. The node exhibiting a DC value higher than twice the median degree of all nodes and achieving the values of the other four indexes over the corresponding median values was defined as a hub [21]. To standardly describe these genes, GO enrichment and KEGG pathway analyses were conducted by using DAVID. Values of *P* < 0.05 were considered exhibiting statistical significance.

### 2.6. Gene Expression Analysis

The pattern of gene expression in lung tissues may indicate that the targets involved in the pathway of asthma development. GSE6858 was used to analyze expression patterns for genes acquired from the GEO database (http://www.ncbi.nlm.nih.gov/geo). In GSE6858, we compared the expression of *Mmp2*, Nr3c1, il-10, il-4, il-13, il-17a, il-2, tlr4, tlr9, ccl2, csf2, and vefg*α* in OVA-stimulated mouse lung tissues compared to the normal group.

## 3. Results

### 3.1. Screening for the Active Component of PT

The 115 reported active ingredients of PT were retrieved from the TCMSP database. Then, the values of OB and DL were employed to screen potential active components, and 13 of the mentioned bioactive ingredients were preliminarily screened out. Besides, only 11 active ingredients with corresponding targets were screened out after the final screening from the PubChem database (Table 1), most of which were sterols (*β*-sitosterol, stigmasterol, and cycloartenol), flavonoids (baicalein and baicalin), unsaturated fatty acid, and lipid, exhibiting high oral bioavailability (ranging from 30.7% to 44.72%) and high drug similarity (0.2∼0.81). Moreover, these ingredients generally displayed the pharmacological activities of antibacterial, anti-inflammatory, and antioxidant.

### 3.2. Component-Target Network Construction and Enrichment Analysis

**Figure 2:**
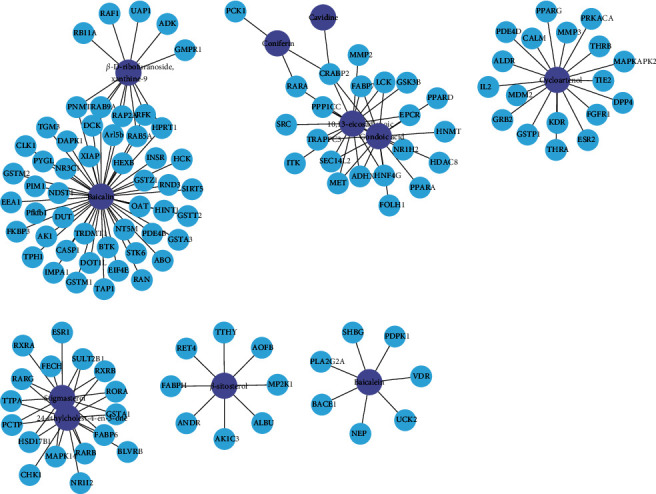
Component-target network (C-T network). The network of 11 active components (the purple nodes) of *Pinellia ternata* and 115 putative targets (the blue nodes) was clustered with Cytoscape version 3.7.2. All protein targets were represented by their gene symbol.

Component-related targets were obtained from the results of the BATMAN-TCM database. Based on the data acquired above, the component-target network was established with Cytoscape 3.7.2 software. After clustering analysis was conducted on the obtained component-target network, six clusters were obtained (Figure 2). Studies have shown that inflammatory responses (immune cell differentiation, cytokine secretion, and receptor signaling) and metabolic responses (drug metabolism and energy metabolism) play an important role in the development of asthma [22]. For example, in the network, cluster 1 includes components baicalin and *β*-D-ribofuranoside, xanthine-9, and their targets which were enriched in signaling pathways of drug metabolism, glutathione metabolism, platinum drug resistance, etc. Cluster 2 includes components cavidine, coniferin, 10,13-eicosadienoic acid, and gondoic acid and their targets, which were enriched in pathways of PPAR signaling pathway and Th17 cell differentiation. Cluster 3 includes component cycloartenol and targets, which exhibit anti-inﬂammatory functions and are associated with pathways of MAPK and PI3K.

### 3.3. Asthma-Related Targets

By searching the OMIM databases, the target data for the treatment of asthma were collected. A total of 259 targets were screened after false-positive information was checked and removed, including cytokines (e.g., TNF, IL-4, IL-5, and IL-33), receptors (e.g., CCR4, IL-6R, and IL-9R), chemokines (e.g., CCL2, CCL11, and CCL18), and transcription factors (e.g., STAT4, STAT6, and GATA3) (Table 2).

### 3.4. Hub Nodes' Screening and PPI Network Construction

**Figure 3:**
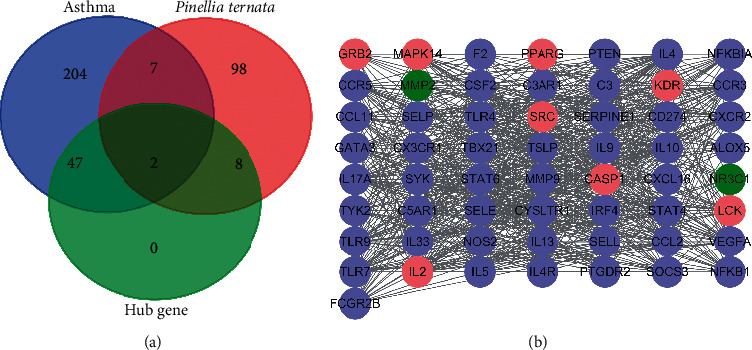
Network of drug and disease targets. (a) The number of asthma-related targets and component targets showed by Venn. (b) Network of hub nodes. The property of the purple nodes is asthma-related targets, the property of the pink nodes is component-related targets, and the property of the green nodes is common targets of asthma and components.

To elucidate the molecular mechanism underlying the effects of PT against asthma, the component-related targets and asthma-related targets were uploaded to STRING to acquire the information on PPI. 365 nodes and 3726 correlations were covered in the network. Next, according to the topology of DC, BC, CC, NC, and LAC, 57 hub nodes and 808 relationships were ascertained. We constructed a PPI network among these 57 hub nodes and presumed those targets as the putative targets of PT for the treatment of asthma (Figures 3(a) and 3(b)).

### 3.5. Hub Nodes' Enrichment Analysis

**Figure 4:**
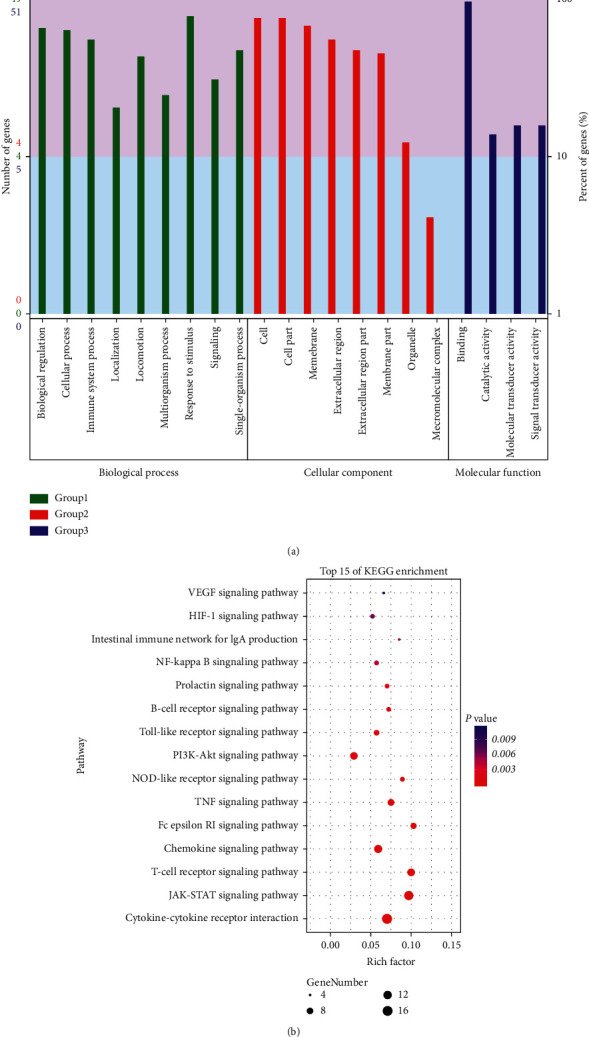
Enrichment analysis of the hub genes for *Pinellia ternata* in the treatment of asthma. (a) The GO enrichment analysis of hub nodes. (b) The top 15 KEGG pathway enrichment analyses of hub nodes.

To identify relevant pathways and functions, GO functional analysis and KEGG pathway enrichment analysis were performed for these 57 putative targets using DAVID. A total of 276 enrichment results were obtained, covering 226 biological processes (BP), 32 molecular functions (MF), and 18 cellular components (CC); the enriched molecular functions of the target proteins are mainly associated with response to stimulus, biological regulation, and cellular process (Figure 4(a)). As revealed from the KEGG enrichment analysis, the signaling pathways were notably enriched in pathways of T-cell receptor signaling pathway, JAK-STAT signaling pathway, and cytokine-cytokine receptor interaction. After extensive pathways were excluded, the top 15 relevant signaling pathways are presented in Figure 4(b). Values of *P* < 0.05 were considered exhibiting statistical significance; the lower the *P* value is, the more prominent the relevance will be.

### 3.6. Expression Profiles of Key Targets in Asthma

**Figure 5:**
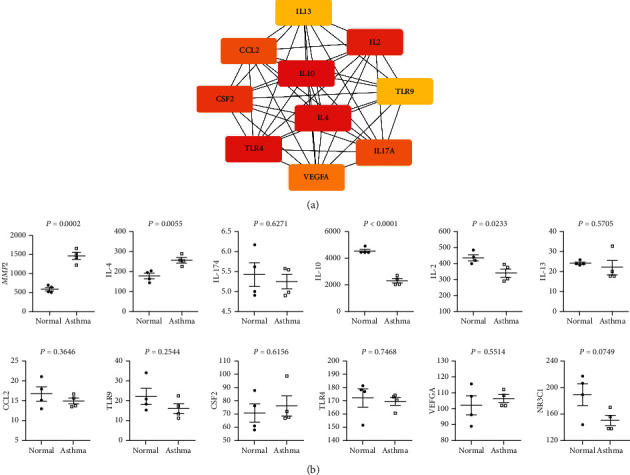
Expression levels of key nodes in lung tissues of allergic mice. (a) The top 10 hub nodes showed by Cytoscape. (b) Gene expression levels of lung tissues in GSE6858 from the GEO database.

Based on the above data, genes matrix metalloproteinase-2 (*Mmp2*) and nuclear receptor subfamily 3, group C, member 1 (Nr3c1) are both component target and highly relevant targets for asthma. il-10, il-4, il-13, il-17a, il-2, tlr4, tlr9, ccl2, csf2, and vegf*α* are top 10 hub nodes in the PPI network (Figure 5(a)), so we inquired the expression profiles of these genes in asthma from the GEO database. In GSE6858, the expression level of *Mmp2* and il-4 in OVA-stimulated mice was upregulated than these of the control group (*P* < 0.001); il-10 and il-2 were downregulated in allergic mice, and there was no significant difference in the expression levels of other genes (Figure 5(b)).

### 3.7. PT Ameliorated OVA-Induced Asthma via Downregulating the Mmp2 and Il-4 Expressions

**Figure 6:**
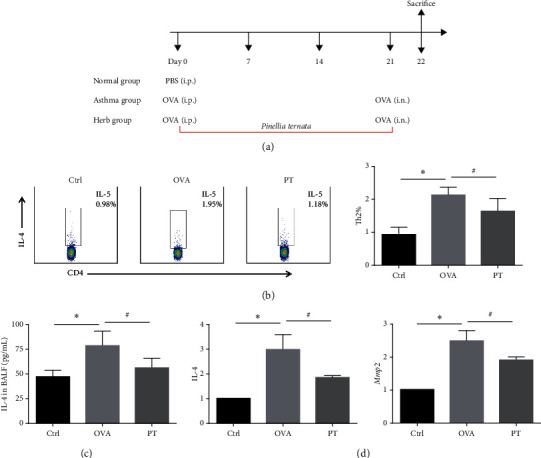
PT ameliorated OVA-induced asthma. (a) The timeline of administration in animal experiments. (b) The percentages of TH2 cells (CD45^+^, CD3^+^, CD4^+^, and IL4^+^) in lymphocytes by FACS. (c) The concentration of IL-4 in BALF. (d) The mRNA levels of il-4 and *Mmp2* in the lung by RT-qPCR. Data shown were normalized to the reference gene GAPDH. Values were expressed as mean ± SEM. ^*∗*^*P* value <0.05 compared to the control group; ^#^*P* value <0.05 compared to the model group.

Asthma is a chronic allergic respiratory disease. Here, we validated the anti-inflammatory and antiasthmatic therapeutic properties of PT, using a mouse model of OVA-induced allergic asthma; the timeline of administration with OVA or PT in mice is shown in Figure 6(a). Unquestionably, OVA-sensitized mice model exhibited an enhanced type 2 immune response as the percentage of TH2 cells in the lung was elevated compared with that of PBS-sensitized control mice (*∗P* < 0.05); in the case of drug treatment, the percentage of TH2 cells was noticeably reversed with PT decoction therapy (#*P* < 0.05) (Figure 6(b)). As suggested by ELISA analysis, the concentration of il-4 was significantly upregulated by OVA treatment as compared with the control group, whereas PT treatment led to the downregulation of il-4 in the BALF of allergic mice (Figure 6(c)). The mRNA levels of *Mmp2* and il-4 were decreased in drug treatment mice compared to OVA-sensitized mice (Figure 6(d)).

## 4. Discussion

The existing number of patients with bronchial asthma worldwide reaches over 300 million, and considerable patients still face difficulty in mitigating their symptoms after undergoing conventional atomization therapy, thereby developing refractory bronchial asthma [23]. The mechanism of asthma pathogenesis remains unclear. Over the past years, numerous researchers have considered that airway inflammation cytokines exhibit abnormally high level on the pathogenesis of asthma and play a critical role in the process. To be specific, IL-4 is capable of stimulating the B lymphocyte to secret large specific IgE, which induces gathered eosinophils and increased airway mucosa, even airway hyperresponsiveness. IL-5 could control the differentiation, maturation, migration, and survival of eosinophils. IL-10 is capable of effectively reducing the secretion activity of mast cells and TH2 cells and exerts a definite effect on the reduction of airway inflammation. Both IL-13 and IL-17 are critical proinflammatory cytokines in the body, thereby facilitating the synthesis of IgE, elevating the level of vascular adhesion molecules, and increasing the degree of eosinophil infiltration in the airway [2, 24]. All of these genes are included in the asthma-related targets (Table 2). Besides, obesity, metabolic disease, and age can also act as risk factors exacerbating asthma [25]. As impacted by heterogeneous in terms of severity, individual difference, and treatment responsiveness, the diverseness of this disease results in multiple therapeutic strategies. Several studies have reported that PT possesses anti-inflammatory and antiallergic effects both in asthma and in COPD [26, 27]. However, the physiological and pharmacological actions of PT on asthma and its biological function have not been effectively studied. The incorporation of traditional Chinese medicine into clinical therapy via network pharmacology can provide insights into the possible mechanism and enhance the specificity and effectiveness of the treatment scheme [5].

In our study, active ingredients were screened in PT, primarily ﬂavonoids, sterols, glycosides, and unsaturated fatty acid, and proteins which were considered as both potential targets of ingredients and targets of asthma treatment were predicted. In the PPI network of component targets and asthma-related targets, hub nodes were screened. Based on the GO terms, the activity of hub nodes was associated with numerous biologic processes (e.g., inflammatory response, chemotaxis, and immune response), a variety of molecular functions (e.g., cytokine activity, growth factor activity, and protein tyrosine kinase activity), and diversified cellular components (e.g., extracellular space, extracellular region, and external side of the plasma membrane). Thus, the results demonstrated that PT primarily exerted various therapeutic effects on asthma by participating in these processes and functions. As further revealed from the KEGG enrichment analysis, the JAK-STAT signaling pathway is one of a handful of pleiotropic cascades and plays essential roles during development and cellular processes (e.g., segmentation, proliferation, and organogenesis) [28]. JAK kinases could selectively phosphorylate STATs, leading to their activation. To be specific, STAT6 acted as one of the primary members in the JAK-STAT pathway, capable of specifically inducing TH2 cell differentiation and promoting the secretion of IL-4 [29]. Our flow cytometry analysis revealed that, in the allergic mice model, PT could attenuate allergic immune response by inhibiting the activation of TH2 cells and the mRNA levels of IL-4. However, the regulation of IL-4 is depended on the way of direct binding to an upstream distal promoter element or on the way of direct activation of the transcription factors such as STAT6 which needed further study.

Notably, *Mmp2* and Nr3c1 are two hub nodes, which are members of both component targets and asthma targets, making them crucial, accounting for PT in asthma treatment. Mmps are a family of enzymes characterized by a common zinc ion at their active site. In general, Mmps are thought to be involved in the normal maintenance of the extracellular matrix and inflammation and cell-cell signaling [30]. Studies reported that the main function of *Mmp2* protease is to degrade extracellular matrix proteins and participate in airway remodeling in asthma [31]. Using *Mmp2*-cleavable activatable cell-penetrating peptides (ACPPs) with the cleavage sequence PLGC (Me) AG, it is shown that *Mmp2* is upregulated in the lungs of allergen OVA-challenged mice [32]; this result is consistent with findings in Figure 5(b). Besides, Nr3c1 is capable of impacting the number of the glucocorticoid receptor (GR) and the affinity with glucocorticoid [33]. The stress hormone cortisol binds to glucocorticoid receptors produced by Nr3c1, resulting in reduced inflammation [34]. Reduced expression of Nr3c1 may lead to glucocorticoid resistance, which gives rise to the inflammatory response [35]. Actually, many individuals with asthma rely on corticosteroid medications to control asthma symptoms [36]. However, in animal experiments, Nr3c1 apparently revealed a large gap in lung tissues of normal mice and ubiquitously expressed lower in most allergic mice lung tissues (Figure 5(b)); this is the reason why we did not test it in subsequent animal studies.

## 5. Conclusions

TCM has been commonly used to treat various diseases and exhibits favorable efficacy, whereas the molecular mechanism of TCM has not been elucidated. In the present study, the network pharmacology method was used to study the scientific basis of PT in the treatment of asthma. 57 hub nodes were screened, and signaling pathways associated with asthma were enriched; these data lay a scientific basis for the clinical treatment of asthma and also provide insights into the development and utilization of drugs. In animal experiments, PT could alleviate the allergic response of mice by inhibiting the activation of TH2 cells and the expression of IL-4 and *Mmp2*; furthermore, systematic and rigorous experiments are required to verify our findings.

## Figures and Tables

**Table 1:** Active components and ADME parameters of *Pinellia ternata*.

PubChem ID	Molecule name	OB	DL
5484202	24-Ethylcholest-4-en-3-one	36.08	0.76
193148	Cavidine	35.64	0.81
5281605	Baicalein	33.52	0.21
64982	Baicalin	40.12	0.75
222284	*β*-Sitosterol	36.91	0.75
5280794	Stigmasterol	43.83	0.76
5282768	Gondoic acid	30.7	0.2
389888	Coniferin	31.11	0.32
5365687	10,13-Eicosadienoic acid	39.99	0.2
92110	Cycloartenol	38.69	0.78
64959	*β*-D-Ribofuranoside, xanthine-9	44.72	0.21

ADME: absorption, distribution, metabolism, and excretion; OB: oral bioavailability; DL: drug similarity.

**Table 2:** Related targets of asthma.

PDE4D	HLA-G	IL6R	TNFRSF4	TPSB2	CD274	ADORA2B	PTGIR
HNMT	ADAM33	TYRO3	SERPINB3	VEGFA	IL25	TACR2	CFB
VDR	PLA2G7	MMP12	SERPINB4	SUV39H1	PDCD1LG2	CYP26A1	TACR3
MMP2	IRAK3	GABRA2	FKBP5	HRH1	TPSD1	TRPV1	CTSK
BTK	C5	GAD2	CPN1	FABP4	IL31	TOP2A	CTSL
NR1I2	CCL11	HAVCR1	ADCY9	STAT4	NKAIN2	ROS1	CTSB
ADH5	LTC4S	MUC7	CCL18	MUC5B	JAML	SELE	CTSS
PDE4B	C5AR1	MARCKS	CLCA1	IL11RA	ORMDL1	CXCR2	AOC3
NR3C1	FLG	GABRB2	C3AR1	CX3CR1	ORMDL2	PDE3A	CCR4
NPSR1	IL12B	TPT1	RASGRP4	IL12RB1	TAS2R31	SELENOP	NOCT
IL13	ORMDL3	CCR5	SETDB2	CCR6	ACTG2	SELL	CNOT6
TNF	SCGB1A1	PTEN	POSTN	JAG1	CLC	ADRB2	TLR9
IL4R	TBX21	DAP3	DENND1B	SATB1	MIF	MCL1	UTS2R
IL9	HLA-DRB1	TLR4	CRHR1	JAG2	IL17F	F2	TSLP
ALOX5	HLA-DRB1	GAD1	CD74	CXADR	MYL3	ADRB3	CSF2RB
PTGDR	HLA-DRB1	TNIP1	FCAR	H3C1	ABCA1	PDE4A	PTGER4
TBXA2R	SCGB3A2	C3	ITGB6	CST7	CAV3	HR	TLR7
PTGDR2	MMP9	FCER1A	IL11	ALOX15B	HPS1	ESRRA	NOS2
CYSLTR1	IL13RA1	PRKCA	ALOX15	FFAR2	ALG9	ITGA5	NANOS2
IL4	CHI3L1	IRF4	LTA	KCNMB1	CDHR3	CCL2	SERPINE1
IL5	CMA1	TNFSF10	TRAF5	SIGLEC5	IL5RA	PRSS1	CBR1
IL13RA2	MS4A2	SOCS3	CFTR	CBX5	ANGPT2	PLA2G1B	ITGA4
ALOX5AP	IL9R	TNFSF14	MUC5AC	ICOS	CHRNA7	HAL	KCNMA1
CYSLTR2	CHIA	SART1	SNCA	FCGR2B	ANGPT1	SYK	MAP3K9
SELP	GATA3	BPIFA1	NFKB1	CD209	MC4R	TACR1	ADRA2C
PTAFR	RUNX3	SPRED1	NFKB2	TAS2R14	IL17A	MICU1	BDKRB2
PTGER3	CCL24	GLCCI1	PTGER2	TAS2R10	IL17RA	SCN9A	OPN4
CCR3	SPINK5	USP38	PTPN6	PDE11A	CHRM4	SCN10A	PDE5A
LTB4R2	NOD1	IL10	SPRR2A	HDAC3	CHRM2	TBXAS1	CSF2
IL33	PHF11	LGALS3	SPRR2B	KCNMB2	CHRM3	LTB4R	ADRB1
PLA2G4A	DPP10	NFKBIA	TNC	LY96	CAMP	ACKR3	CHRM5
STAT6	HSD11B2	SERPINB2	TPSAB1	CXCL16	CHAMP1	ADORA1	MME
ATP2A2	ACE	TYK2					

## Data Availability

The datasets used and/or analyzed during the current study are available from the corresponding author upon reasonable request.

## Abbreviations

TH2 cells: T-helper type 2 cells
IL-2: Interleukin-2
GM-CSF: Granulocyte macrophage colony-stimulating factor
ICS: Inhaled corticosteroid
TCM: Traditional Chinese medicine
PT: *Pinellia ternata*
OB: Oral bioavailability
DL: Drug similarity
GO: Gene ontology
KEGG: Kyoto Encyclopedia of Genes and Genomes
OMIM: Online Mendelian Inheritance in Man database
DC: Degree of centricity
BC: Betweenness centrality
CC: Close to the central
NC: Network centricity
LAC: Local edge connectivity topology selection
SPF: Specific pathogen-free
OVA: Ovalbumin
BALF: Bronchoalveolar lavage fluid
BP: Biological processes
MF: Molecular functions
CC: Cellular components
MMP2: Matrix metalloproteinase-2
NR3C1: Nuclear receptor subfamily 3, group C, member 1
ACPPs: Activatable cell-penetrating peptides
GR: Glucocorticoid receptor.
